# Epipolar Rectification with Minimum Perspective Distortion for Oblique Images

**DOI:** 10.3390/s16111870

**Published:** 2016-11-07

**Authors:** Jianchen Liu, Bingxuan Guo, Wanshou Jiang, Weishu Gong, Xiongwu Xiao

**Affiliations:** 1 School of Remote Sensing and Information Engineering, Wuhan University, 129 Luoyu Road, Wuhan 430079, China; liujianchen@whu.edu.cn; 2 State key Laboratory for Information Engineering in Surveying, Mapping and Remote Sensing, Wuhan University, 129 Luoyu Road, Wuhan 430079, China; jws@whu.edu.cn (W.J.); xwxiao@whu.edu.cn (X.X.); 3 Department of Geographical Sciences, University of Maryland, 2181 Samuel J. LeFrak Hall, 7251 Preinkert Drive, College Park, MD 20742, USA; weishugong@gmail.com

**Keywords:** epipolar rectification, oblique images, UAV images, minimum perspective distortion, 3D reconstruction

## Abstract

Epipolar rectification is of great importance for 3D modeling by using UAV (Unmanned Aerial Vehicle) images; however, the existing methods seldom consider the perspective distortion relative to surface planes. Therefore, an algorithm for the rectification of oblique images is proposed and implemented in detail. The basic principle is to minimize the rectified images’ perspective distortion relative to the reference planes. First, this minimization problem is formulated as a cost function that is constructed by the tangent value of angle deformation; second, it provides a great deal of flexibility on using different reference planes, such as roofs and the façades of buildings, to generate rectified images. Furthermore, a reasonable scale is acquired according to the dihedral angle between the rectified image plane and the original image plane. The low-quality regions of oblique images are cropped out according to the distortion size. Experimental results revealed that the proposed rectification method can result in improved matching precision (Semi-global dense matching). The matching precision is increased by about 30% for roofs and increased by just 1% for façades, while the façades are not parallel to the baseline. In another designed experiment, the selected façades are parallel to the baseline, the matching precision has a great improvement for façades, by an average of 22%. This fully proves our proposed algorithm that elimination of perspective distortion on rectified images can significantly improve the accuracy of dense matching.

## 1. Introduction

Aerial oblique imagery has become an important source for acquiring information about urban areas because of their visualization, high efficiency and wide application in domains such as 3D modeling, large-scale mapping and emergency relief planning. An important characteristic of oblique images is the big tilt angles [1], and they usually contain large perspective distortions relative to the surfaces. This large distortion reduces the image correlations and makes dense image matching more difficult, so traditional techniques usually perform poorly on oblique images. However, the precise 3D reconstruction tasks require an accurate dense disparity map, e.g., using a SGM (Semi-global Matching) based stereo method [2], therefore, epipolar rectification is a necessary initial step for 3D modeling [3]. To guarantee completeness, robustness and precision, image rectification for the purpose of 3D reconstruction should take the perspective distortion into account.

This paper is inspired by the fact that epipolar rectification can minimize perspective distortion; thus, features can be matched very accurately by correlation and an accurate dense disparity map can be generated [4]. Each urban scene usually contains multiple surface planes, and these planes can be grouped according to their feature directions (horizontal or vertical). The epipolar rectification should minimize the distortion of planes in the same group to create an exact match for those planes. The epipolar rectification principle of minimum perspective distortion relative to the original image is desirable in the vast majority of cases; however, rectified images with a minimum perspective distortion relative to surface planes are also useful in certain circumstances. For example, in oblique photogrammetry, rectified horizontal images are usually used to reconstruct the roofs of buildings, meanwhile rectified vertical images are expected to generate more accurate depth maps of vertical planes such as the façades of buildings.

Many epipolar rectification algorithms have been proposed, and they can generally be categorized into linear transformation and non-linear transformation algorithms. The former algorithms transform an image from one plane to another plane, making the corresponding epipolar lines coincide with the scan lines [5]. The linear approaches’ advantages are that it is mathematically simple, fast and preserves image features. In contrast, non-linear approaches typically use Bresenham’s algorithm [6] to extract pixels along epipolar lines, thus avoiding most of the problems that linear rectification approaches have, e.g., generating unbounded, large or badly warped images. Two similar methods [7,8] involve parameterizing the image with polar coordinates (around the epipoles). These methods have two important features: they can address epipoles located in the images, and they can reduce the matching ambiguity to half the epipolar lines. Instead of resampling the epipolar lines on the original image planes, an attractive method proposed by [9] employs a rectification process based on a cylinder rather than a plane. However, this method is relatively complex and requires large numbers of calculations in three-dimensional space. Another non-linear transformation method expressed in a paper by [10] proposes an accurate computation method based on rectification of spherical-camera images via resampling the same longitude line. However, this method is suitable only for spherical panoramic images.

Because linear transformation is simple and intuitive, this article focuses on the homography based method. Due to the redundant degrees of freedom, the solution to rectification is not unique and can actually lead to undesirable distortions. The distortion constraint leads to reduced degrees of freedom for homographies in solving the rectification problem. The first work on using a distortion constraint was performed by [11], followed by [12]. They suggest using the transformation that minimizes the range of disparity between the two images, i.e., the distance between the rectified corresponding points. In their state-of-the-art methods [13,14], the authors attempt to make the effects of rectification “as affine as possible” over the area of the images. In papers by [15], a different distortion criterion consists of preserving the sampling of the original images. The method proposed in the paper [16] uses the squared Sampson error for the constrained rectification. The algorithm [17] is decomposed into three steps: the first and second step involve making the image plane parallel to the baseline, while the third crucial step minimizes the distortion relative to the original images. None of the above methods mention the fact that there is no agreement on what the distortion criterion should be, and they all require corresponding points. In a paper by [18], the authors aim to eliminate relative distortion between the rectified images by choosing the reference plane in the scene. However, only the reference plane and the planes that are parallel to the reference plane have no relative distortion and their method still requires the corresponding points. In the case of calibrated cameras, the rotation matrix for rectified images is determined directly. The method [19] determines the rectified image plane according to the baseline and the optical axis of the old left matrix; however, in that case, the distortion for one of the rectified images is small, but the distortion of the other may be larger in the oblique photography case. The algorithm expressed in the paper [20] improves the preceding algorithm by making the distortion of the two rectified images small relative to the original images. However, these methods still do not consider distortion relative to surfaces in object space.

Unlike the methods described above, which reduce distortion by explicitly minimizing an empirical measure, the proposed approach is to minimize a cost function that is constructed by the tangent value of angle deformation. In this manner, the rectified images will have smallest perspective distortion for some surface planes and features can be matched quite accurately by correlation. In addition, the homography based method may yield very large images or cannot rectify at all. These issues can be solved by the scope constraint which can also crop the low-quality regions of oblique images.

In this paper, we investigated the rectification method of minimum perspective distortion by taking into account surface planes, such as original image planes, roofs and the façades of buildings. The method is flexible in order to generate rectified images with respect to different reference planes. The remainder of this paper is organized as follows. The innovative rectification algorithms and their distortion constraints are presented in detail in Section 2. The performance of the proposed methods and the quantitative evaluation of the matching results are subsequently evaluated in Section 3. Finally, concluding remarks are provided.

## 2. Methodology

### 2.1. Algorithm Principle

**Figure 1:**
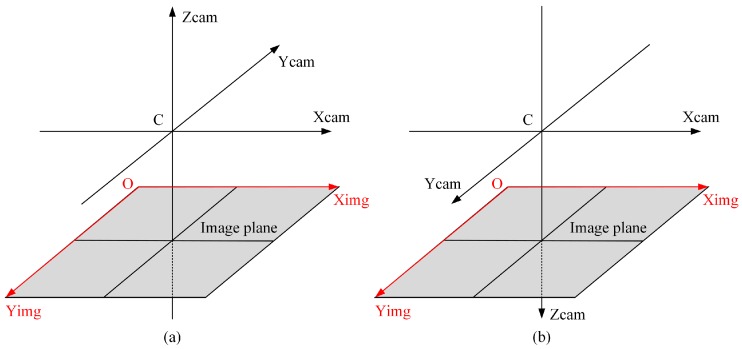
The camera coordinate frames for (**a**) photogrammetry definition; (**b**) computer vision definition.

There is little difference between computer vision (CV) and photogrammetry (DP) in terms of definitions of projective geometry. The projective matrix P generated by both the computer vision and photogrammetry definitions is the same. However, the expressions of the camera matrix *K* and the rotation matrix *R* are different. This is because they define the camera coordinate frame differently [21], which is shown in Figure 1.

The origin of coordinates C and coordinate axes $X_{cam},Y_{cam},Z_{cam}$ constitute the camera coordinate frame. The image coordinate system is consisted of the origin of coordinates O and coordinate axes $X_{img},Y_{img}$. From Figure 1, we can see that the camera coordinate frames are both right hand Euclidean coordinate systems. However, the image plane is Z=f in computer vision and Z=−f in photogrammetry, where f>0. The relationship between $R_{CV}$ and $R_{DP}$ is shown below:

(1) $$R_{CV}=\left[\begin{array}{ccc} 1 & 0 & 0\\ 0 & -1 & 0\\ 0 & 0 & -1 \end{array}\right]R_{DP}$$

As per the different camera coordinate frames, the camera calibration matrix *K* can be respectively denoted as:

(2) $$K_{CV}=\left[\begin{array}{ccc} f_{x} & 0 & x_{0}\\ 0 & f_{y} & y_{0}\\ 0 & 0 & 1 \end{array}\right]\;K_{DP}=\left[\begin{array}{ccc} -f_{x} & 0 & x_{0}\\ 0 & f_{y} & y_{0}\\ 0 & 0 & 1 \end{array}\right]\;K_{CV}=-K_{DP}\left[\begin{array}{ccc} 1 & 0 & 0\\ 0 & -1 & 0\\ 0 & 0 & -1 \end{array}\right]$$

where $f_{x},f_{y}$ represents the focal length of the camera in terms of pixel dimensions in the *x* and *y* direction, respectively. The expression $\left(x_{0},y_{0}\right)$ is the principal point in terms of pixel dimensions. In this article, the symbols *K* and *R* refer to the definition used by the photogrammetry field. Thus, the direction of the image plane and the *Z* axis of the camera coordinate frame defined in photogrammetry are in accord. This selection is more convenient for the subsequent rectifying transformation when considering the perspective distortion relative to reference planes.

#### 2.1.1. Homographic Transformation

Epipolar rectification can be viewed as the process of transforming the epipolar geometry of a pair of images into a canonical form. It can be accomplished by applying a homographic matrix to each image that maps the original image to a predetermined plane. Let H and $H^{\prime }$ be the homographic matrix to be applied to images I and $I^{\prime }$, respectively. Also, let p∈I and $p^{\prime }\in I^{\prime }$ be a pair of corresponding points. The camera matrix $K_{rec}$ and the rotation matrix $R_{rec}$ can be generated by the algorithm proposed in this paper, while the symbols *R* and *K* refer to the original image. Considering the rectified image points $p_{rec}\in I_{rec}$, $p_{rec}^{\prime }\in I_{rec}^{\prime }$, the transformation can be defined as:

(3) $$\begin{array}{l} p_{rec}=Hp\\ p_{rec}^{\prime }=H^{\prime }p^{\prime } \end{array}$$

(4) $$\begin{array}{l} H=K_{rec}R_{rec}R^{T}K^{-1}\\ H^{\prime }=K_{rec}^{\prime }R_{rec}^{\prime }R^{\prime T}K^{\prime -1} \end{array}$$

However, there are countless types of transformation matrices H that meet the above conditions of the solution. Moreover, poor choices for H and $H^{\prime }$ can result in rectified images that are dramatically changed in scale or severely distorted. Therefore, rectified image planes should be selected according to the criteria of minimum perspective distortion, and it will be discussed in the next section.

#### 2.1.2. Minimizing Perspective Distortion

The angle deformation always exists in the perspective transformation from a reference configuration to a current configuration. The scale of rectified images can be determined by the focal length, and it does not affect the angle deformation. In the process of rectification, a method is developed to minimize the tangent value of angle deformation. The subscript L and R denote the left and right images respectively in the following of the paper. Here, it can be defined as:

(5) $$\varepsilon =(\omega_{L}{)}^{2}+{\left(\omega_{R}\right)}^{2}$$

where ω is the tangent value of angle deformation. The result can be determined by minimizing the squared error ε. The angle deformation presents a notable positive correlation with the rotation angle, i.e., the dihedral angle between the rectified image plane and its reference plane (original image plane or surface plane). It is easy to discuss the characteristics of angle deformation by decomposing it into two directions: the rotation direction and its perpendicular direction. A line in reference plane which is perpendicular to the rotation direction has no angle deformation, while the angle deformation of a line that is parallel to the rotation direction could not be ignored. The relationship of the rotation angle θ with the tangent value of angle deformation is given below:

(6) ω=−sin(θ)⋅b

in which b determines the position of a line in the reference plane. Thus, Equation (5) can be rewritten as:

(7) $$\varepsilon =(\sin(\theta_{L}{))}^{2}+{\left(\sin(\theta_{R})\right)}^{2}$$

**Figure 2:**
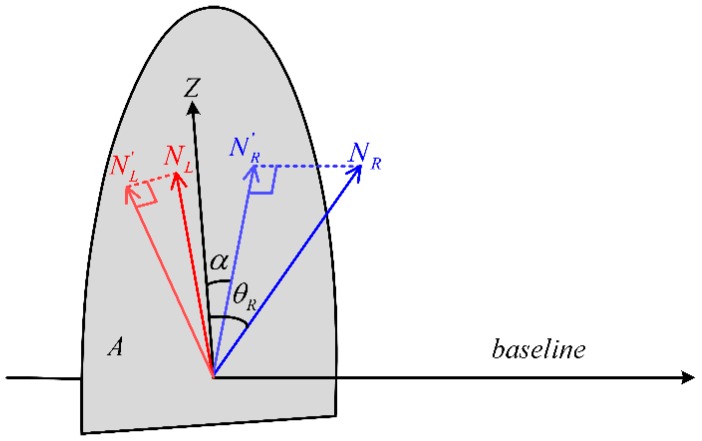
The rectification of minimum perspective distortion.

According to the principle of epipolar rectification, the two rectified image planes ($I_{rec}$ and $I_{rec}^{\prime }$) must be corrected to be coplanar, and must both be parallel to the baseline (B). Thus, the direction Z of the rectified image plane is constrained to be perpendicular to the baseline, which lies in a plane A perpendicular to the baseline. In the case that left and right reference planes are different, the rectification of minimum perspective distortion is illustrated in Figure 2. $N_{L}$ and $N_{R}$ are the directions of reference planes. Their projections on a plane A are $N_{L}^{\prime }$ and $N_{R}^{\prime }$. The α in Figure 2 denotes the angle between $N_{R}^{\prime }$ and Z. Thus $\theta_{L}$ and $\theta_{R}$ can be expressed respectively by a function that takes one parameter α, and these expressions can be easily derived by the analytic geometry. Furthermore, the direction Z of rectified image plane is determined by one parameter α. The solution is to minimize the squared error ε by gradient descent method. In the case that left and right reference planes are the same, the direction Z of rectified image plane is the projection of the direction vector N onto the plane A.

### 2.2. Rectification Algorithm

#### 2.2.1. *R* Matrix of Rectified Image

After expressing the observational coordinate axes of the camera coordinate frame numerically as three unit vectors $\left(e_{0},e_{1},e_{2}\right)$ in the world coordinate system, together they comprise the rows of the rotation matrix *R* (world to camera). The rectified images with respect to different reference planes are controlled by the *R* matrix. The *R* matrix calculation is simple and flexible as explained in the following sections.

##### Basic Rectification

A minimum distortion rectification relative to the original image planes is discussed first and it can be applied to a variety of cases. To carry out this method, it is important to construct a triple of mutually orthogonal unit vectors $\left(e_{1},e_{2},e_{3}\right)$. The first vector $e_{1}$ can be given by the baseline. Because the baseline is parallel to the rectified image plane and the epipolar line is horizontal, vector $e_{1}$ coincides with the direction of the baseline. $C_{1},C_{2}$ are the camera station coordinates and $e_{1}$ can be deduced as:

(8) $$e_{1}=\frac{B}{\left\|B\right\|},\;B=C_{2}-C_{1}$$

The two constraints on the second vector, $e_{2}$, are that it must be orthogonal to $e_{1}$ and that the perspective distortion relative to the original images must both be minimal. To achieve these, it should compute and normalize the cross product of $e_{1}$ with $e_{temp}$, which is the direction Z of rectified image plane (see Section 2.1.2). It can be expressed as:

(9) $$e_{2}=\frac{e_{temp}\times e_{1}}{\left\|e_{temp}\times e_{1}\right\|}$$

The third unit vector is unambiguously determined as:

(10) $$e_{3}=e_{1}\times e_{2}$$

Together, they comprise the rows of the rotation matrix R, which is defined as:

(11) $$R_{rec}=\left[\begin{array}{c} e_{1}\\ e_{2}\\ e_{3} \end{array}\right]$$

Thus, the rectified camera coordinate frames are defined by getting the *R* matrix of the rectified images. Noting that the left and right *R* matrices are same.

##### Horizontal or Vertical Rectification

When the image models are absolutely oriented, horizontally or vertically rectified images can be generated. At the same time, it can minimize the perspective distortion relative to horizontal or vertical planes, making the result conducive for image-matching purposes for regular buildings. Because the baseline is not absolutely horizontal, the way to minimize the perspective distortion relative to horizontal planes is to generate the rectified images that are closest to the horizontal plane. However, absolutely rectified vertical images can be generated according to Section 2.1.2. The computational process is similar to the above procedures. There is only a slight difference in the definition of $e_{temp}$. For horizontal images, it is defined as follows:

(12) $$e_{temp}=\left[\begin{array}{ccc} 0 & 0 & 1 \end{array}\right]$$

When vertical images are needed, the $e_{temp}$ should meet the following constraints:

1. $e_{temp}=\left[\begin{array}{ccc} x & y & 0 \end{array}\right]$;
2. $e_{temp}$ must be orthogonal to the baseline;
3. $e_{temp}$ should be consistent with the two direction vectors of the original images’ optical axes, i.e., $e_{temp}\cdot R_{3}>0,\;e_{temp}\cdot R_{3}^{\prime }>0$.

##### General Rectification

When the images models are relatively oriented or when a non-horizontal or non-vertical plane exists in the world coordinates, the direction of the rectified image planes should be closest to the direction of the plane to minimize the distortion with respect to that plane. The computational process is similar to the above two procedures, requiring only a small difference in the definition of $e_{temp}$. Given a plane expressed as $\left[\begin{array}{cccc} a & b & c & d \end{array}\right]$, its normal form is expressed as $\left[\begin{array}{ccc} a & b & c \end{array}\right]$, which is consistent with the two direction vectors of the original images’ optical axes. Then, $e_{temp}$ is determined as:

(13) $$e_{temp}=\left[\begin{array}{ccc} a & b & c \end{array}\right]$$

From the above discussion, it is easy to obtain various rectified images with different distortion characteristics by defining different *R* matrices, which works because the definition of the *R* matrix is flexible.

#### 2.2.2. Camera Matrix of Rectified Image

**Figure 3:**
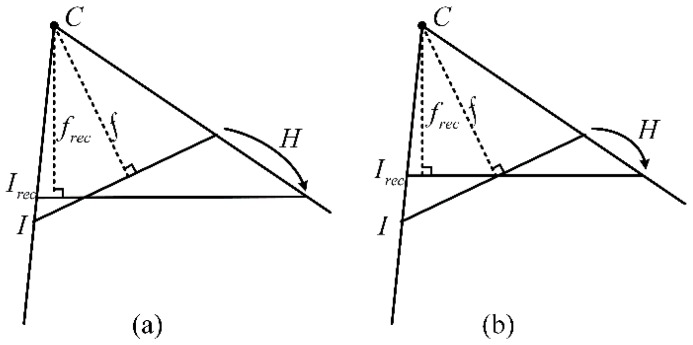
The two methods for setting focal length: (**a**) the focal length unchanged; (**b**) the principle point unchanged.

The scale of both rectified images can be adjusted by setting a suitable focal length. As we know, the focal lengths of rectified images have the same value. The most commonly used method, shown in Figure 3a, is to set the focal length the same as the original images; however, in that case, the rectified images will be larger than the original images. Note that although the resolution is higher than in the original images, it is meaningless. Our method, shown in Figure 3b, is to keep the principle point of the original image unchanged during the perspective transformation.

In Figure 3a, the rectified images will be larger than the original images. In particular, as the rotation angle between the optical axes of the original and rectified images grows larger, the rectified image size becomes significantly bigger. In contrast, in Figure 3b, the rectified image size would not be significantly different from the original image. The proposed method may result in rectified images in which some part of the image is compressed and the other part is stretched compared to the original images. However, the average resolution remains almost unchanged from the original images. In this paper, the focal length is defined as:

(14) $$\begin{array}{l} f_{1}=f\cdot R_{3}\cdot e_{3}\\ f_{2}=f^{\prime }\cdot R_{3}^{\prime }\cdot e_{3}\\ f_{rec}=\min(f_{1},f_{2}) \end{array}$$

After getting the *R* matrix and the focal length of the rectified images, it is easy to obtain the *K* matrices of the rectified images. According to Section 2.1.1, the *H* matrices can be calculated to rectify the original images to rectified images.

### 2.3. Distortion Constraints

#### 2.3.1. Distortion Coordinate Frame

To better express the character of distortion relative to the original images, a distortion coordinate frame is defined. The optical axis of the rectified image is the $Z_{dis}$ axis of the distortion coordinate frame, i.e., the third row of the rectified rotation matrix $R_{rec}$. The $X_{dis}$ and $Y_{dis}$ axes of this coordinate frame can be obtained by the cross products. The $Y_{dis}$ axis must be orthogonal to the two optical axes of the original image and rectified image, and can be expressed as:

(15) $$Y_{dis}=R_{3}\times R_{rec3}$$

The third unit vector is unambiguously determined as:

(16) $$X_{dis}=Y_{dis}\times Z_{dis}$$

The three unit vectors of $X_{dis},Y_{dis},Z_{dis}$ form a rotation matrix $R_{dis}$.

#### 2.3.2. Characteristics of Distortion

**Figure 4:**
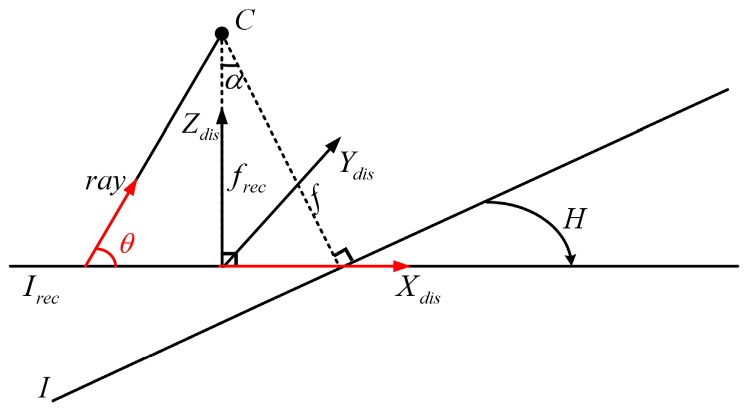
The distortion coordinate frame.

**Figure 5:**
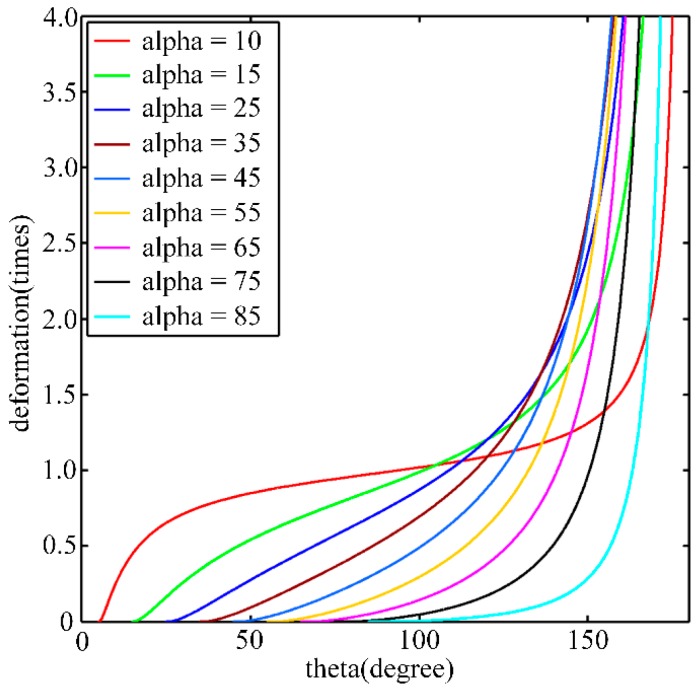
The curves of distortion relative to original image.

This section focus on the distortion of rectified image relative to the original image. It is easy to discuss the characteristics of distortion in the distortion coordinate frame. The distortion within an image line that is parallel to the $Y_{dis}$ axis has the same size, while the distortion within an image line that is parallel to the $X_{dis}$ axis gradually becomes larger along the positive direction of the $X_{dis}$ axis. The size of distortion (denoted as t) is the ratio between the size of a point in the original image and the size of its corresponding point in the referencing image. It is derived from the projection geometry and shown in Equation (17), a schematic diagram is introduced in Figure 4:

(17) $$t=\cos\alpha {\left(\cos\alpha -\sin\alpha \cot\theta \right)}^{2}$$

where α is the angle between the Z axes of the original and the rectified camera coordinate frames. Assume that the field of view (FOV) of the original image is π, although the FOV is usually less than that value in actuality. For the rectified image, the valid FOV range is (α,π) and θ∈(α,π). The θ in Figure 4 denotes the angle from the directional vector $X_{dis}$ to the ray of light. The size of the distortion is closely related to the angle α. Its characteristics are illustrated in Figure 5.

Figure 5 shows nine curves that correspond to different α values. The horizontal axis represents the ray direction θ, and the vertical axis represents the image distortion relative to original image. Through the distribution curve of distortion, a curve that is far away from the line y=1 corresponds to an image with large distortion. It can be observed that the greater the angle α is, the greater the corresponding distortion is. Moreover, the distortion near the image edges is greater than the distortion near the image center. For the images used in this paper, the tilt angle of oblique images is approximately 45°, and the field of view is approximately 35°–50°, so rectified images typically do not have such large distortions.

#### 2.3.3. Constraint Method

The size of the maximum distortion can constrain the scope of the image and can be applied to the following two aspects: constraining the unbounded images and getting the highest quality image region. When generating the rectified horizontal images, distortion constraints can remove the image region with the smaller base-to-height ratio and the image areas that are likely to be blurred due to atmospheric influence. In oblique aerial photography, the upper part of the image is the region with the smaller base-to-height ratio and is also highly likely to be affected by air quality, making it blurry.

If the rotation angle α is large and the FOV of original image is also large, it is likely to generate an unbounded image. This phenomenon is most likely to appear in close range photogrammetry and oblique photogrammetry. Given the threshold (the size of maximum distortion) T, thus Equation (17) can be rewritten as:

(18) $$\tan\theta =\frac{\sin\alpha }{\cos\alpha -\sqrt{\frac{T}{\cos\alpha }}}$$

Equation (18) can provide the result of calculating the desirable image region. Then, translating the coordinates of the constrained image scope from the distortion coordinate frame to the rectified camera coordinate frame $\left(R_{dis}\to R_{rec}\right)$. Finally, solving for the intersection area of the constrained image scope (solved by Equation (18) under a threshold *T*) and the original image’s projective scope (calculated by Equation (3) using image 4 corner points) in the rectified image plane for the final scope of the rectified image.

## 3. Experimental Evaluations

### 3.1. Performance of Rectification

The presented approach is tested with oblique images captured by the SWDC-5 aerial multi-angle photography system. This system is composed of five large format digital cameras with one vertical angle and four tilt angles. The image size of the five cameras is 8176 × 6132, and the pixel size is 6 µ. The angles of the four tilt cameras are 45° relative to the vertical camera. The focal length of the tilt cameras is 80 mm, while the focal length of the vertical camera is 50 mm. The relative height of flight is 1000 m possessing a GSD (Ground Sampling Distance) of 12 cm. The side and forward overlapping rates are 50% and 80% respectively. The coordinates are recorded in the WGS-84 coordinate system. Oblique photography captures more information, including the façade textures of the buildings, which can be used to create a more realistic appearance in 3D urban scene modelling.

When reconstructing 3D architectures from oblique images, calibration is mandatory in practice and can be achieved in many situations and by several algorithms [22,23]. Given a pair of stereo oriented images, the corresponding P (projection matrix), or the intrinsic parameters of each camera and the extrinsic parameters of the images, it is straightforward to define a rectifying transformation. Meanwhile, it is needed to minimize the perspective distortion according to the above methods. In this article, the lens distortions are not considered and have already been removed in the experimental data.

**Figure 6:**
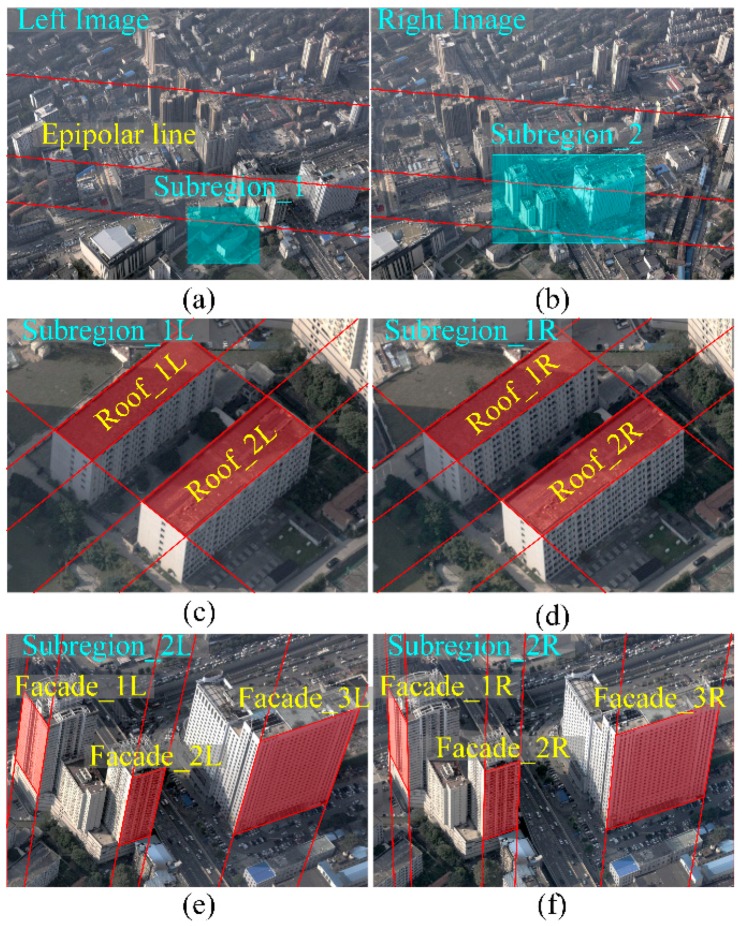
The original image pair and its sub-regions: the red lines in (**a**,**b**) are the epipolar lines in the original image; the red areas in (**c**,**d**) are the horizontal roofs projected in the original images; the red areas in (**e**,**f**) are vertical façades projected in the original images.

**Figure 7:**
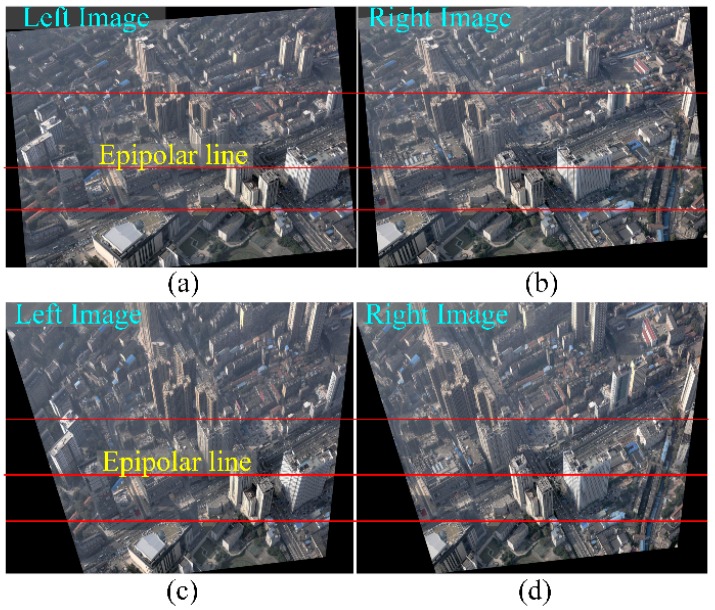
The result of epipolar rectification: (**a**,**b**) show the rectified image pair with the minimum distortion relative to original images; (**c**,**d**) show the horizontally rectified image pair with a scope constraint.

**Figure 8:**
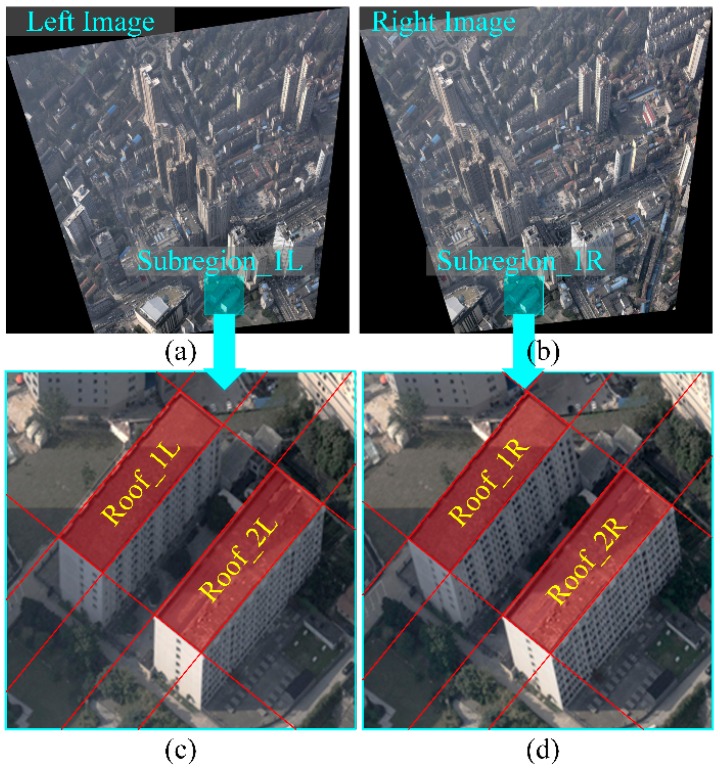
A rectified horizontal image pair (**a**,**b**); and their sub-regions (**c**,**d**), in which the shapes of horizontal planes projected in the rectified images are similar.

**Figure 9:**
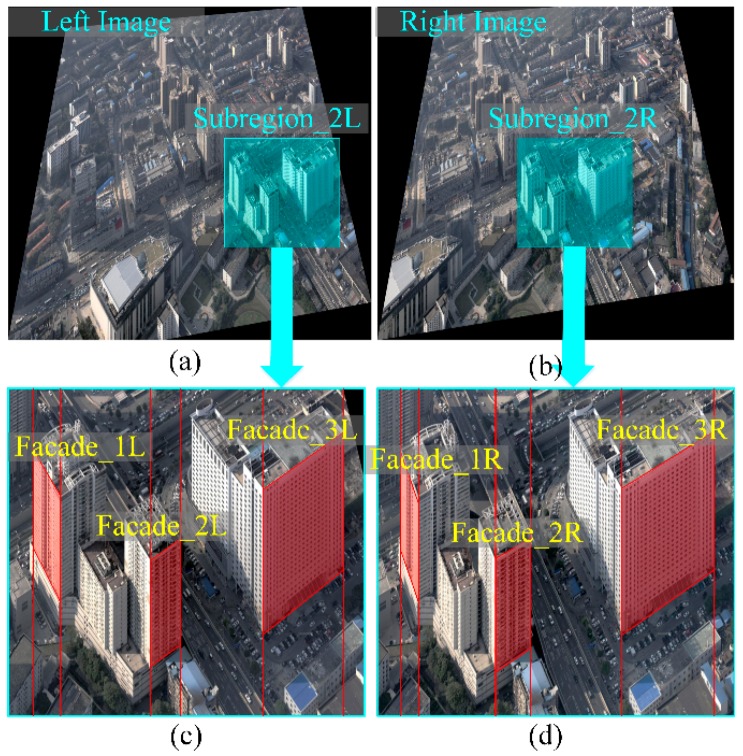
The rectified vertical image pair (**a**,**b**); and their sub-regions (**c**,**d**), in which the vertical lines projected into the rectified images are true vertical lines.

Figure 6a,b shows the original image pair captured from Wuhan City (China) in which the red lines are epipolar lines. In the sub-region pair in Figure 6c,d, the roofs are shown with perspective distortion, which is especially apparent in Figure 6e,f with the building façades. Examples of rectified image pairs illustrate basic rectification (Figure 7a,b), horizontal rectification (Figure 8), vertical rectification (Figure 9) and scope-constrained rectification (Figure 7c,d). Figure 7a,b shows the rectified image pair with minimum distortion properties relative to the original images, i.e., the changes to the optical axis are minimal. It is apparent that the epipolar lines (red lines) are horizontal in the rectified images and that the corresponding lines are in nearly the same vertical position. Figure 8a,b shows a horizontally rectified image pair while Figure 8c,d shows their sub-regions in which the roofs (red areas) are similar and without distortion, i.e., the disparities are close to a constant. There is no distortion for the horizontal objects projected into the horizontally rectified images, but absolutely rectified horizontal images do not exist for the non-horizontal baseline. Although there is a slight distortion for the horizontal objects in this type of rectification, the distortion is minimal and can be ignored for oblique aerial photography. Using a small adjustment, images without the distortion of horizontal objects can be achieved by setting different focal lengths and making the rectified image plane absolutely horizontal. However, the method cannot generate rectified images in this way. Figure 9 clarifies the concepts of vertically rectified images. In that figure, the vertical lines of façades are still vertical in the vertically rectified images as shown in Figure 9c,d compared to Figure 6e,f. Typically, there is no distortion for façades in the vertical direction, while in the horizontal direction, scale distortion is inevitable unless the façades are all parallel to the vertically rectified image plane. From these results, the façades can be considered for flight course planning. Figure 7c,d shows the rectification result under the scope constraint. Due to the large tilt angle, the image regions that have a smaller base-to-height ratio are removed as can be observed in Figure 7c,d compared to Figure 8a,b. This method can also be used to constrain the unbounded rectified image area, which happens when the tilt angle is large and the field of view of is also large, as in oblique photogrammetry.

### 3.2. Quantitative Evaluation of the Matching Results

In this section, the matching results of commonly used rectifications [19,20] and the proposed rectification are comparatively analyzed. These two commonly used rectifications are very similar to the proposed basic rectification. In addition, there are small differences for their dense matching results, which can be ignored for the dataset used in this paper. However, the commonly used methods do not consider the perspective distortion relative to surfaces in object space. Therefore, there are dramatic differences compared to the proposed horizontal and vertical rectification. The following quantitative analysis shows the superiority of the proposed rectification method.

**Figure 10:**
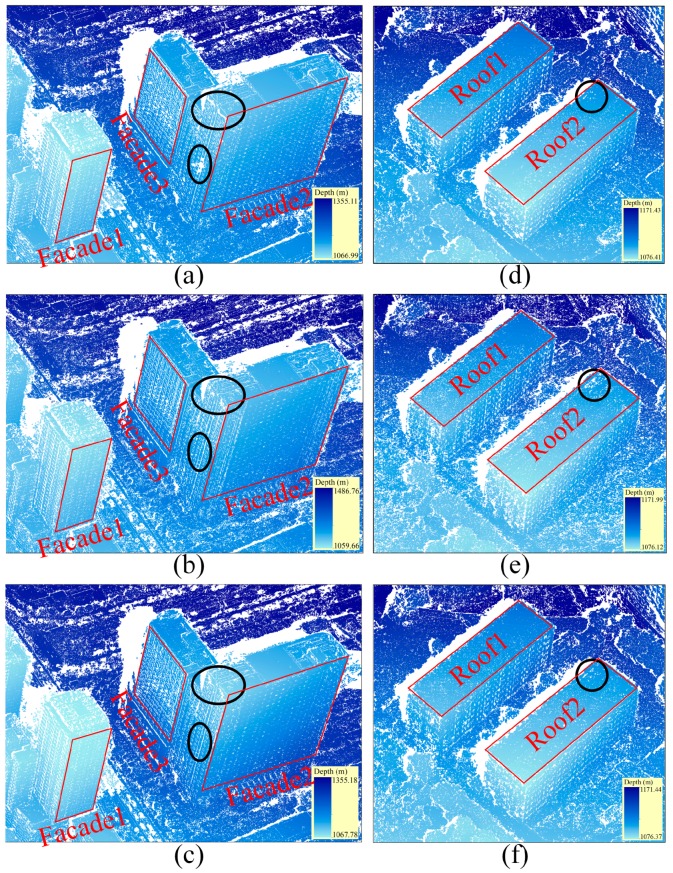
A comparison of matching results for roofs and façades: (**a**,**d**) the matching result of rectified horizontal images; (**b**,**e**) the matching result of rectified vertical images; (**c**,**f**) the matching result of commonly used rectification images.

Here, we select horizontal roofs and vertical façades (red areas shown in Figure 10) to evaluate the matching precision influenced by the distortions. Three sets of rectified images pairs were matched by the tSGM algorithm [24] and the resulting depth maps were evaluated quantitatively. For the horizontal rectification, the roofs appear to be without perspective distortion, however, the distortions of façades are not eliminated. In contrast, the distortions of façades are minimized in the vertical rectification, which is opposite to the roofs. For the commonly used rectification, the roofs and the façades are both with geometry distortion. All dense image matches were carried out on full resolution imagery. For comparison purposes, the resulting depth maps have been transformed from rectified images to original images.

The matching results of horizontal objects are compared in Figure 10d–f, showing that the densities of point clouds in the roof areas are not the same, especially in the black area. The horizontally rectified image (matching result shown in Figure 10d) generates more points than the vertical rectification (matching result shown in Figure 10e) and commonly used rectification (matching result shown in Figure 10f) for the roofs. The matching result for the façades in Figure 10a–c shows that the vertical rectification is the best as expected. Vertical rectification is more convenient for matching façades and generates a denser set of points than the other rectifications. To differentiate them more convincingly, the result of a quantitative analysis is first shown in Table 1. The influence of deformation on matching can be analyzed from two aspects: the percentage of valid pixels and the precision. The former means the percentage of generated depth pixels within an area. In the latter case, the RMSE (Root Mean Square Error) of plane fitting is used to scale the precision of image matching. From Table 1, we can see that due to the reduced size of the distortions, the density of point clouds increases from 98.89% to 99.15%, and the RMSE is reduced from 15.2 cm to 10.1 cm for Roof 1 (the red area in Figure 10). Similar changes occurred for Roof 2. Because of the high matching result from tSGM, the precision is obviously improved, though there is no significant improvement in the integrity of roofs. Using the same analytical method for façades, both increased integrity and precision for vertical rectification are shown in Table 2, but not as obviously as for the roofs. This is probably due to the fact that the façades are not parallel to the baseline, and the scale deformation in the horizontal direction still exists in vertical rectification. Nevertheless, the rectification methods proposed in this paper can improve the matching precision. The matching results of horizontal and vertical rectifications are compared in Table 3. It shows that the matching results of horizontal objects in horizontal rectification are better than that of the vertical rectification, while there are almost completely opposite conclusions for façades. In the case of roofs, experimental data also show that the matching results of horizontal rectification perform the best for precision, because the distortion of roofs in horizontal rectification is smallest in these three situations. A similar conclusion can be drawn for façades.

### 3.3. Robustness Evaluation

We choose another set of data captured from Nanchang City (China) to evaluate the robustness of the proposed algorithm. The image data is captured by a multi-angle oblique photography system composed of five large format digital cameras: one vertical angle and four tilt angles. The image size of vertical view is 9334 × 6000 and the image size of tilt views is 7312 × 5474. The pixel size is 6 µ. The angle of four tilt cameras is 45° relative to the vertical camera. The focal length of tilt cameras is 80 mm and that of vertical camera is 50 mm. The relative height of flight is 1000 m. The distance of adjacent strips is 500 m and that of adjacent images within the same strip is 200 m.

**Figure 11:**
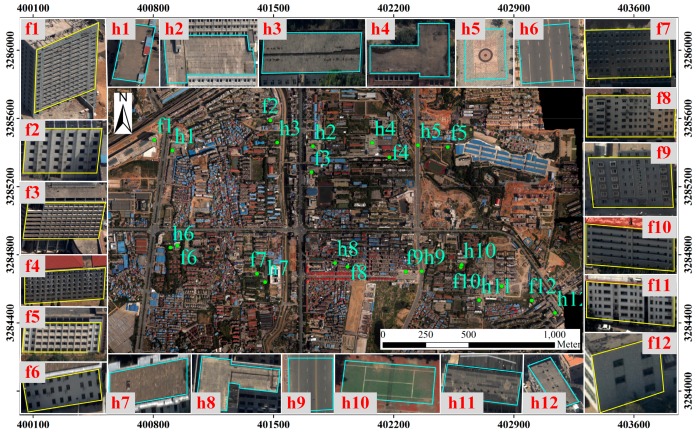
The illustration of coverage area and selected test planes. We choose 12 façades and 12 horizontal planes (including roofs, playgrounds and roads) within the coverage area. In the orthoimages, the plane positions are marked. The selected planes are shown in the surroundings.

Three trips with a total of 210 images are used in the experiment and cover an area of 7 km^2^. Coverage area is a city district, and there are a lot of horizontal and vertical planes. Images are processed through bundle adjustment, automatic DEM (Digital Elevation Model) extraction and orthoimage production steps with the GodWork software package (version 2.0), which has been developed by Wuhan University (Wuhan, China). We choose 12 façades and 12 horizontal planes (including roofs, playgrounds and roads) within the coverage area, which are shown in Figure 11. The selected planes are evaluated by using different oriented image pairs.

We use the same method as mentioned in Section 3.2 to evaluate the matching precision influenced by the distortions. Table 4 shows the evaluation results of the horizontal planes. It shows that the matching precision is increased by about 33% for horizontal planes. The analysis results are in agreement with the Section 3.2. From Table 5, we can see that the matching precision in vertical rectification also shows a great improvement for façades, by an average of 22%. The analysis results are significantly different from Section 3.2. This is within our expectations. Because the flight direction is from east to west, it is easier to select a number of façades parallel to the baseline. Thus, there is no distortion for the façades projected into the vertically rectified images. This fully proves our hypothesis that perspective distortion has a great influence on matching. Elimination of perspective distortion on rectified images can significantly improve the accuracy of dense matching.

## 4. Conclusions

Epipolar rectification does not usually take into account the distortions of surface planes and the quality of original images. Therefore, a new rectification algorithm for aerial oblique images is proposed that minimizes the distortion of surface planes. The method is based on the minimization of a cost function that is constructed by the tangent value of angle deformation. In addition, a scope-constrained rectification is proposed to solve the problems of unbounded rectified images and crop out the low-quality areas of oblique images. Although the method proposed in this paper seems simple, it addresses epipolar rectification of oblique images in a flexible manner and solves many practical problems in oblique image matching.

The proposed strategy of epipolar rectification leads to depth maps with greater numbers of valid pixels and increased precision by minimizing the perspective distortion. The experiments have confirmed that the matching precision for horizontal objects can be significantly improved by using the proposed rectification method (increased by about 30%). This improvement is attributed to the fact that the horizontal objects appear to be without distortions in the horizontal rectification. However, the distortions of façades have not been completely eliminated, and scale deformation in the horizontal direction is inevitable unless the façades are parallel to the baseline. Therefore, the façade directions should be considered for flight course planning. In a second set of data, the flight direction is from east to west, and most of the visible façades are parallel to the baseline. In this condition, the matching precision shows a great improvement for façades, by an average of 22%. This fully proves that perspective distortion has a great influence on matching. Elimination of perspective distortion on rectified images can significantly improve the accuracy of dense matching. Furthermore, a better result could be achieved by integrating two depth maps of horizontal rectification and vertical rectification in 3D modeling.

## Figures and Tables

**Table 1:** A comparison of matching results for horizontally rectified images and commonly used rectification images.

	Objects	Horizontal Rectification		Commonly Used Methods	
		Integrity (%)	Precision (RMSE)	Integrity (%)	Precision (RMSE)
**Horizontal Objects**	**Roof 1**	99.15%	10.1 cm	98.89%	15.2 cm
	**Roof 2**	98.86%	18.1 cm	98.55%	21.5 cm

**Table 2:** A comparison of matching results for vertically rectified images and commonly used rectification images.

	Objects	Commonly Used Methods		Vertical Rectification	
		Integrity (%)	Precision (RMSE)	Integrity (%)	Precision (RMSE)
**Vertical Façades**	**Façade 1**	92.12%	53.3 cm	92.47%	52.5 cm
	**Façade 2**	97.45%	24.7 cm	98.52%	24.5cm
	**Façade 3**	79.28%	27.1 cm	82.40%	25.6 cm

**Table 3:** A comparison of matching results for horizontally rectified images and vertically rectified images.

	Objects	Horizontal Rectification		Vertical Rectification	
		Integrity (%)	Precision (RMSE)	Integrity (%)	Precision (RMSE)
**Horizontal Objects**	**Roof 1**	99.15%	10.1 cm	97.94%	22.4 cm
	**Roof 2**	98.86%	18.1 cm	97.31%	27.2 cm
**Vertical Façades**	**Façade 1**	91.44%	56.6 cm	92.47%	52.5 cm
	**Façade 2**	97.18%	30.4 cm	98.52%	24.5cm
	**Façade 3**	78.63%	27.6 cm	82.40%	25.6 cm

**Table 4:** A comparison of matching results for horizontally rectified images and commonly used rectification images.

Objects	Commonly Used Methods Precision (RMSE)	Horizontal Rectification Precision (RMSE)	Improvement (%)
**h1**	20.02 cm	15.30 cm	23.56%
**h2**	15.81 cm	9.57 cm	39.46%
**h3**	14.98 cm	8.14 cm	45.69%
**h4**	10.08 cm	6.55 cm	35.00%
**h5**	39.75 cm	25.59 cm	35.62%
**h6**	8.92 cm	6.34 cm	28.91%
**h7**	20.50 cm	13.78 cm	32.80%
**h8**	13.82 cm	7.64 cm	44.75%
**h9**	37.63 cm	27.41 cm	27.16%
**h10**	33.30 cm	20.93 cm	37.15%
**h11**	17.99 cm	11.26 cm	37.41%
**h12**	28.20 cm	24.95 cm	11.54%

**Table 5:** A comparison of matching results for vertically rectified images and commonly used rectification images.

Objects	Commonly Used Methods Precision (RMSE)	Vertical Rectification Precision (RMSE)	Improvement (%)
**f1**	17.18 cm	15.05 cm	12.42%
**f2**	22.18 cm	18.25 cm	17.72%
**f3**	32.88 cm	25.12 cm	23.58%
**f4**	44.15 cm	36.67 cm	16.94%
**f5**	17.02 cm	8.95 cm	47.39%
**f6**	30.72 cm	22.92 cm	25.38%
**f7**	23.71 cm	14.46 cm	39.02%
**f8**	39.87 cm	35.09 cm	12.00%
**f9**	27.83 cm	21.18 cm	23.90%
**f10**	41.20 cm	32.79 cm	20.40%
**f11**	44.21 cm	32.42 cm	26.68%
**f12**	50.84 cm	47.12 cm	7.31%

## References

[1] Peters J. (2015). A look at making real-imagery 3D scenes (texture mapping with nadir and oblique aerial imagery). Photogramm. Eng. Remote Sens..

[2] Heiko H. (2008). Stereo processing by semiglobal matching and mutual information. IEEE Trans. Pattern Anal. Mach. Intell..

[3] Shahbazi M., Sohn G., Théau J., Menard P. (2015). Development and evaluation of a UAV-photogrammetry system for precise 3D environmental modeling. Sensors.

[4] Kim J.-I., Kim T. (2016). Comparison of computer vision and photogrammetric approaches for epipolar resampling of image sequence. Sensors.

[5] Sun C. Trinocular stereo image rectification in closed-form only using fundamental matrices. Proceedings of the 2013 20th IEEE International Conference on Image Processing (ICIP).

[6] Chen Z., Wu C., Tsui H.T. (2003). A new image rectification algorithm. Pattern Recognit. Lett..

[7] Oram D. Rectification for any epipolar geometry. Proceedings of the British Machine Vision Conference.

[8] Pollefeys M., Koch R., Van Gool L. A simple and efficient rectification method for general motion. Proceedings of the Seventh IEEE International Conference on Computer Vision.

[9] Roy S., Meunier J., Cox I.J. Cylindrical rectification to minimize epipolar distortion. Proceedings of 1997 IEEE Computer Society Conference on Computer Vision and Pattern Recognition (CVPR’97).

[10] Fujiki J., Torii A., Akaho S. (2007). Epipolar Geometry via Rectification of Spherical Images.

[11] Hartley R., Gupta R. Computing matched-epipolar projections. Proceedings of the IEEE Computer Society Conference on Computer Vision and Pattern Recognition.

[12] Hartley R.I. (1999). Theory and practice of projective rectification. Int. J. Comput. Vis..

[13] Loop C., Zhang Z. (1999). Computing Rectifying Homographies for Stereo Vision.

[14] Wu H.H.P., Yu Y.H. (2005). Projective rectification with reduced geometric distortion for stereo vision and stereoscopic video. J. Intell. Robot. Syst..

[15] Mallon J., Whelan P.F. (2005). Projective rectification from the fundamental matrix. Image Vis. Comput..

[16] Fusiello A., Irsara L. (2011). Quasi-euclidean epipolar rectification of uncalibrated images. Mach. Vis. Appl..

[17] Monasse P., Morel J.M., Tang Z. (2010). Three-Step Image Rectification.

[18] Al-Zahrani A., Ipson S.S., Haigh J.G.B. (2004). Applications of a direct algorithm for the rectification of uncalibrated images. Inf. Sci..

[19] Fusiello A., Trucco E., Verri A. (2000). A compact algorithm for rectification of stereo pairs. Mach. Vis. Appl..

[20] Kang Y.S., Ho Y.S. (2011). Efficient Stereo Image Rectification Method Using Horizontal Baseline.

[21] Wang H., Shen S., Lu X. Comparison of the camera calibration between photogrammetry and computer vision. Proceedings of the International Conference on System Science and Engineering.

[22] Kedzierski M., Delis P. (2016). Fast orientation of video images of buildings acquired from a UAV without stabilization. Sensors.

[23] Triggs B., Mclauchlan P.F., Hartley R.I., Fitzgibbon A.W. (1999). Bundle Adjustment—A Modern Synthesis.

[24] Rothermel M., Wenzel K., Fritsch D., Haala N. Sure: Photogrammetric surface reconstruction from imagery. Proceedings of the LC3D Workshop.

